# 血栓性血小板减少性紫癜诊断与治疗中国指南（2022年版）

**DOI:** 10.3760/cma.j.issn.0253-2727.2022.01.002

**Published:** 2022-01

**Authors:** 中华医学会血液学分会血栓与止血学组

一、概述

血栓性血小板减少性紫癜（thrombotic thrombocytopenic purpura, TTP）为一种少见、严重的血栓性微血管病，其主要临床特征包括微血管病性溶血性贫血（MAHA）、血小板减少、神经精神症状、发热和肾脏受累等[1]。TTP的发病机制主要涉及血管性血友病因子（VWF）裂解酶（ADAMTS13）活性缺乏，也与血管内皮细胞VWF异常释放、补体异常活化、血小板异常活化等相关。血浆中ADAMTS13活性缺乏导致内皮细胞异常释放的超大分子VWF（UL-VWF）不能及时降解，UL-VWF可自发结合血小板，导致微血管内血栓形成、微血管病性溶血，进而引起相应器官缺血、缺氧及功能障碍，引起临床症候群[2]–[5]。

根据ADAMTS13缺乏机制不同，TTP分为遗传性TTP（cTTP，又称为Upshaw-Schulman综合征）和免疫性TTP（iTTP）。cTTP系ADAMTS13基因突变导致血浆ADAMTS13活性缺乏，常在感染、炎症或妊娠等促发因素下发病。cTTP呈常染色体隐性遗传，基因突变表现为纯合子型或双重杂合子型。iTTP系因患者体内产生抗ADAMTS13自身抗体，抑制ADAMTS13活性（中和抗体）或与ADAMTS13结合形成抗原抗体复合物而加速ADAMTS13在体内清除。iTTP多无明确原因（即原发性），也可能继发于感染、药物、肿瘤、自身免疫性疾病、造血干细胞移植等。iTTP是最常见的临床类型，约占TTP总例数的95％；cTTP较为少见，仅占总例数的5％，但在儿童和孕妇患者中cTTP却占到25％～50％[2]。

二、临床表现

国外资料显示，TTP年发病率为2～6/百万，女性与男性之比约为2∶1，高峰发病年龄为30～50岁，但部分cTTP患者在新生儿期即可发病；多数TTP患者发病急骤、病情危重，少数患者发病隐匿、临床表现不典型；炎症、感染、妊娠等是诱发TTP常见的原因，女性cTTP患者常在妊娠早期出现疾病发作[6]–[7]。

TTP典型临床表现如下：①出血：以皮肤、黏膜为主，严重者可有内脏或颅内出血。②MAHA：多为轻、中度贫血，可伴黄疸。③神经精神症状：表现为意识紊乱、头痛、失语、惊厥、视力障碍、谵妄、偏瘫以及局灶性感觉或运动障碍等，缺乏典型表现，以发作性、多变性为特点。④肾脏损害：可出现蛋白尿、血尿、管型尿，血尿素氮及肌酐轻度升高。⑤发热（>37.5 °C）。⑥胸痛、腹痛、乏力、关节痛、肌肉痛等其他器官损伤的临床表现。

临床上完全符合TTP典型“五联征”的患者相对少见，以MAHA、血小板减少和神经精神症状为主的“三联征”为多见。由于部分TTP患者神经精神症状不显著，建议如发现MAHA和血小板减少时，就要高度警惕TTP可能，及时进行相关实验室检查和全面临床评估。

三、实验室检查

1. 血常规及血涂片检查：不同程度贫血，外周血涂片可见破碎红细胞（>1％），网织红细胞比例大多增高；血小板计数显著降低（多低于20×10^9^/L），且动态下降较显著。

2. 血生化检查：主要有血胆红素升高，以间接胆红素升高为主；血清乳酸脱氢酶（LDH）明显升高；血尿素氮及肌酐不同程度升高，肌钙蛋白T水平升高见于心肌受损者。

3. 血浆ADAMTS13活性及抑制物或IgG抗体测定：目前多采用FRET-VWF荧光底物测定法、残余胶原结合试验或免疫性ELISA方法测定ADAMTS13活性、ADAMTS13抑制物或IgG抗体。TTP患者血浆ADAMTS13活性显著降低（<10％）；iTTP患者ADAMTS13活性显著降低且检出ADAMTS13抑制物或IgG抗体；cTTP患者不存在ADAMTS13抑制物或IgG抗体，基因测序有助于鉴别诊断。血浆ADAMTS13活性及抑制物或IgG抗体测定血样尽可能在血浆置换前留取，同时注意高胆红素血症、高脂血症、游离血红蛋白升高、血浆蛋白酶可能干扰血浆ADAMTS13活性检测，在分析结果时需要注意。

4. 凝血检查：活化部分凝血活酶时间（APTT）、凝血酶原时间（PT）及纤维蛋白原检测多正常，偶有纤维蛋白降解产物轻度升高。

5. 溶血相关检查：红细胞直接抗人球蛋白试验阴性，但在部分继发于免疫性疾病的患者中可为阳性；血浆游离血红蛋白增加、血清结合珠蛋白下降。

6. ADAMTS13基因检测：对怀疑cTTP患者可进行ADAMTS13基因突变检测，有助于确立诊断及遗传咨询。

7. 其他：乙型肝炎病毒（HBV）、丙型肝炎病毒（HCV）、人类免疫缺陷病毒（HIV）病毒血清学检查，甲状腺功能，抗核抗体谱，狼疮抗凝物，抗磷脂抗体、颅脑CT、磁共振成像（MRI）检查及脑电图。

四、临床诊断

1. 具备TTP临床表现：常有MAHA和血小板减少，并非所有患者均具备所谓的“三联征”或“五联征”，临床上需仔细分析病情、寻找病因。

2. 典型的血细胞变化和血生化改变：贫血、血小板计数显著降低，尤其是外周血涂片中红细胞碎片>1％；血清游离血红蛋白增高，血清乳酸脱氢酶明显升高。

3. 血浆ADAMTS13活性显著降低（<10％）：iTTP者常检出ADAMTS13抑制物或IgG抗体。

4. 排除溶血尿毒综合征（HUS）、弥散性血管内凝血（DIC）、HELLP综合征、Evans综合征、子痫、灾难性抗磷脂抗体综合征等疾病。

临床表现典型的患者诊断不难，但多数患者临床表现存在明显个体差异，部分患者临床表现不具特征性，需结合多方面资料综合判断。对初发患者应全面收集临床资料，对疑似患者需进行TTP发病危险度评估，推荐使用PLASMIC评分系统[8]（表1）：积分0～4分为低危，TTP预测效率<5％；积分5分为中危，预测效率5％～25％；积分6～7分为高危，预测效率60％～80％。临床验证发现评分为高危者诊断TTP的敏感性为81.7％、特异性71.4％[9]。

**表1:** 用于评估血栓性血小板减少性紫癜（TTP）发病危险度的PLASMIC评分表

项目	分值
外周血血小板计数<30×10^9^/L	1
溶血证据（网织红细胞>2.5％、间接胆红素>34.2 µmol/L、结合珠蛋白消失）	1
无进展期癌症	1
无实体器官移植或干细胞移植史	1
平均红细胞体积（MCV）<90 fl	1
凝血酶原时间国际标准化比值（PT-INR）<1.5	1
肌酐<20 mg/L（176.8 µmol/L）	1

对临床评估中度或高度疑似TTP的患者应及时留取血样本送检ADAMTS13活性及抑制物或IgG抗体测定，不必等待检测结果回报即开始血浆置换和糖皮质激素治疗。如后续检测报告血浆ADAMTS13活性<10％正常混合血浆活性，即可诊断TTP；血浆ADAMTS13活性>20％者可基本排除TTP；血浆ADAMTS13活性10％～20％并不能完全排除TTP，需根据临床判断及密切随访[10]。TTP诊断流程见图1。

**图1:**
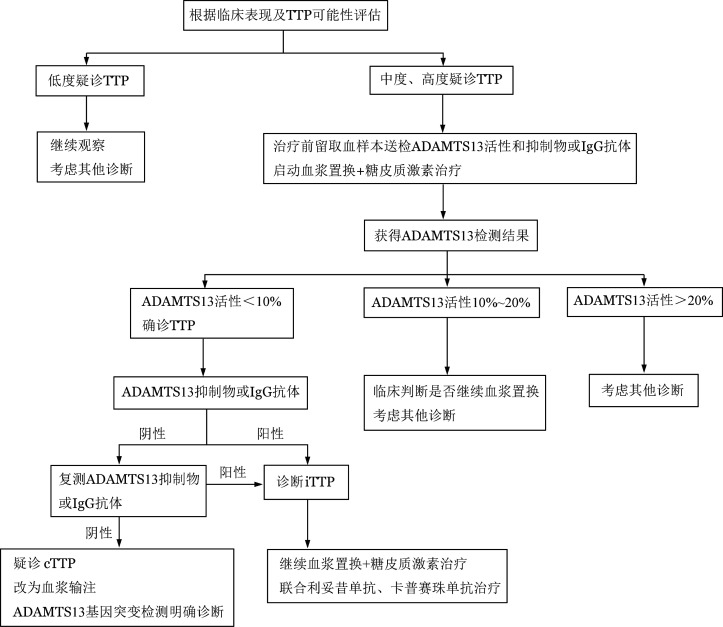
血栓性血小板减少性紫癜（TTP）诊断流程图 ADAMTS13：血管性血友病因子裂解酶；iTTP：免疫性TTP；cTTP：遗传性TTP

五、治疗

（一）治疗原则

本病多急性发病，如不能及时治疗死亡率高。临床上在中度或高度怀疑本病时即应尽快开始相关治疗。iTTP首选血浆置换治疗，并酌情联合使用糖皮质激素等。cTTP以替代治疗为主，分为按需治疗和预防治疗方法。对高度疑似和确诊病例输注血小板应十分谨慎，血浆置换后如出现危及生命的严重出血时才考虑使用[11]。

（二）治疗方法

1. 治疗性血浆置换：适用于iTTP的治疗和临床中/高度怀疑TTP的初始紧急治疗。血浆置换采用新鲜（冰冻）血浆，血浆置换量推荐为每次2000～3000 ml或40～60 ml/kg体重，每日1～2次，直至症状缓解、血小板计数恢复正常连续2 d后可逐渐延长血浆置换间隔直至停止。当肾功能衰竭时，可与血液透析联合应用。血浆置换通过清除血液中ADAMTS13抑制物或IgG抗体及其他致病因素、补充缺乏的ADAMTS13而发挥作用。患者对血浆置换的治疗反应差异较大，对连续血浆置换治疗5次仍未取得临床反应的患者不建议过早停止血浆置换，除继续相关治疗外还应积极寻找诱因（如感染等）并加以祛除。对确无血浆置换条件者，可暂输注新鲜（冰冻）血浆每日20～40 ml/kg。注意液体量平衡。

2. 糖皮质激素：糖皮质激素可减轻炎症反应、保护器官功能、抑制自身抗体产生，主要适用于iTTP治疗。可选用甲泼尼龙（80～120 mg/d）或地塞米松（15～20 mg/d）静脉输注，病情缓解后可过渡至泼尼松（1～2 mg·kg^−1^·d^−1^）并逐渐减量至停用。使用糖皮质激素要考虑到其内分泌、心血管和神经精神系统的不良反应，对伴存高血压、糖尿病、精神疾病及老年患者应特别关注药物的不良反应。

3. 利妥昔单抗（Rituximab）：利妥昔单抗通过选择性耗竭B淋巴细胞而降低ADAMTS13抑制物或IgG抗体滴度，有效恢复血浆ADAMTS13活性。临床研究证实，iTTP急性发作期使用利妥昔单抗可提升治疗有效率、降低早期死亡率、减少复发率、延长缓解期。利妥昔单抗推荐剂量为375 mg/m^2^每周1次，连续应用4周。小剂量利妥昔单抗治疗（100 mg每周1次，连用4周）效果在探索中。建议利妥昔单抗在血浆置换后开始用药，与下次血浆置换间隔20～24 h[11]–[12]。

4. 卡普赛珠单抗（Caplacizumab）：卡普赛珠单抗可阻断VWF A1区与血小板糖蛋白GPⅠb结合作用，阻止血小板-VWF相互作用并防止小动脉和毛细血管内微血栓形成、减少终末器官损害。卡普赛珠单抗在TTP发病早期使用可以最大获益。但卡普赛珠单抗并不能纠正ADAMTS13缺乏，也不能清除ADAMTS13自身抗体。卡普赛珠单抗首次10 mg静脉输注，次日起每日10 mg皮下注射，停止血浆置换后仍需持续使用30 d[11],[13]–[14]。

5. 大剂量静脉免疫球蛋白：治疗iTPP的效果不及血浆置换，仅适用于难治性TTP患者或多次复发的病例。

6. 其他免疫抑制剂：对利妥昔单抗无效或复发的iTTP患者可选用其他免疫抑制剂（硼替佐米、环孢素A等）。硼替佐米通过阻止ADAMTS13自身抗体产生发挥治疗作用，常用剂量为1.3 mg/m^2^皮下注射，每疗程4次（第1、4、8、11天），1～2个疗程。环孢素A常用剂量为3～5 mg·kg^−1^·d^−1^，根据血浆浓度调整剂量。

7. 乙酰半胱氨酸：为还原型谷胱甘肽的前体，可减少多肽链之间的二硫键连接降低VWF多聚化程度，减少组织氧化损伤。在血浆置换后使用有一定的辅助治疗作用。常用剂量8 g/d，缓慢静脉输注[15]。

8. 血小板输注：原则上在高度疑似TTP且尚未进行血浆置换的患者不宜进行血小板输注，因其可能会增加微血栓形成和器官损伤。但在血浆置换后，如出现危及生命的重要器官出血时可考虑进行血小板输注。

9. 预防性血浆输注：适用于cTTP患者的预防性治疗，常用新鲜冰冻血浆每次10～15 ml/kg，输注间隔根据患者血小板数变化情况而定，每1～3周1次。反复输注需注意输血相关疾病传播风险。

10. 重组人ADAMTS13：已进入Ⅲ期临床研究，尤其适合cTTP患者的预防性治疗。

11. 抗血小板药物：iTTP患者病情稳定后可选用潘生丁或阿司匹林，对减少复发有一定作用。

12. 支持治疗：本病累及多个器官，需要及时动态评估各器官功能，给予相应的支持治疗，保护器官功能。

（三）治疗方案及调整

对临床中度或高度疑似或确诊的TTP（尤其是iTTP）患者应立即开始治疗性血浆置换联合糖皮质激素治疗，并可考虑联合卡普赛珠单抗治疗。根据ADAMTS13活性及抑制物或IgG抗体结果调整治疗：如测定的患者血浆ADAMTS13活性<10％且伴抑制物或IgG抗体阳性，符合iTTP则继续进行上述治疗并及时给予利妥昔单抗治疗；如抑制物阴性，考虑cTTP，可停用糖皮质激素、改血浆置换为血浆输注；如患者血浆ADAMTS13活性>20％，则考虑其他诊断并改用相应治疗；血浆ADAMTS13活性10％～20％的患者需根据临床判断是否继续或停止现行治疗。

对复发的iTTP患者，除治疗性血浆置换联合糖皮质激素治疗外，如之前未用过利妥昔单抗或曾使用利妥昔单抗有效但1年后复发者，加用利妥昔单抗治疗。利妥昔单抗后1年内复发的患者可选择其他免疫抑制剂（如硼替佐米、环孢素A）清除ADAMTS13抑制物，恢复ADAMTS13活性。

对缓解期cTTP患者，建议采用血浆输注或密切观察的预防策略，根据患者病情、意愿及可能的不良反应决定治疗选择。对新生儿期发病、有器官损伤的cTTP患者推荐预防治疗。不建议使用血浆源性因子Ⅷ浓缩物因其ADAMTS13含量甚低。重组ADAMTS13将是更为便捷高效的治疗方法。

iTTP女性妊娠时有较高的疾病复发风险，尤其是持续血浆ADAMTS13活性降低者常常是复发先兆，对母体和胎儿均存在不利影响。预防性治疗可能有助于减少母婴死亡率，如iTTP孕妇血浆ADAMTS13活性<10％可进行血浆置换，每周1～2次；如出现TTP临床表现需每日1次血浆置换；联合使用糖皮质激素治疗。孕期不建议使用抗CD20单抗。对上述治疗无效或伴发其他病理产科情况（如妊高症）时需提前终止妊娠。对cTTP的孕妇建议自妊娠开始即进行血浆输注，输注间隔随孕期而逐渐缩短，从每2周1次至隔日1次不等；如出现TTP临床表现，则需增加输注量或改为血浆置换；血浆输注治疗需维持至产后3周。重组ADAMTS13更适合cTTP孕妇的预防治疗。

六、预后

国际TTP工作组最近再次修订了iTTP治疗结局的定义[16]。①临床反应：经血浆置换等治疗后持续血小板计数≥100×10^9^/L和LDH<1.5倍正常值上限，并且无新发器官缺血损伤或原有器官缺血损伤加重。经5次血浆置换治疗仍未取得临床反应者称为难治性TTP。②临床恶化：在取得临床反应后停止血浆置换或抗VWF治疗后30 d内，再次出现血小板计数<100×10^9^/L，伴或不伴有器官缺血损伤再发临床证据。③临床缓解：停止血浆置换或抗VWF治疗30 d后仍能持续维持临床反应者，或取得ADAMTS13缓解者（ADAMTS13部分缓解：ADAMTS13活性≥20％且<正常值下限；ADAMTS13完全缓解：ADAMTS13活性>正常值下限）。④临床复发：在取得临床缓解后，再次出现血小板计数<100×10^9^/L且ADAMTS13活性<10％，伴或不伴有器官缺血损伤临床证据。⑤ADAMTS13复发：在取得ADAMTS13缓解后，再次发生ADAMTS13活性<20％。ADAMTS13复发常发展为临床复发。

iTTP患者在初次发作取得临床缓解后存在复发风险，感染、手术、妊娠等均为诱发因素，而血浆ADAMTS13活性<10％、ADAMTS13抑制物或IgG抗体持续阳性是临床复发的高危因素。所有缓解期的iTTP患者除常规检查血常规外，均应定期复查ADAMTS13活性及其抑制物或IgG抗体，至少在第1年前6个月内每月1次，后6个月内每3个月1次，第二年每6个月1次。随着免疫抑制治疗的早期使用，iTTP复发率有明显减少趋势。cTTP患者在首次发作后常会持续较长时间的病情波动，需要进行预防性治疗；新生儿期发病的cTTP患者常病情严重、器官远期损伤可能性大，需尽早开展预防治疗。

由于TTP发病累及多个器官发生缺血缺氧损伤。TTP临床缓解后神经系统常见的后遗症包括认知障碍、乏力、注意力及记忆力异常等，但并不影响正常工作和活动，部分TTP患者恢复后表现易抑郁和沮丧。也可引起肾脏疾病、心肌损害、高血压等，与器官损伤有关。多学科诊疗团队的合作可以更好地开展患者疾病状态评估和实施相应的治疗。
